# Angiostatic Factors in the Pulmonary Endarterectomy Material from Chronic Thromboembolic Pulmonary Hypertension Patients Cause Endothelial Dysfunction

**DOI:** 10.1371/journal.pone.0043793

**Published:** 2012-08-20

**Authors:** Diana Zabini, Chandran Nagaraj, Elvira Stacher, Irene M. Lang, Patrick Nierlich, Walter Klepetko, Akos Heinemann, Horst Olschewski, Zoltán Bálint, Andrea Olschewski

**Affiliations:** 1 Experimental Anesthesiology, Department of Anesthesia and Intensive Care Medicine, Medical University of Graz, Graz, Austria; 2 Institute of Pathology, Medical University of Graz, Graz, Austria; 3 Department of Internal Medicine II, Division of Cardiology, Medical University of Vienna, Vienna, Austria; 4 Department of Thoracic Surgery, Medical University of Vienna, Vienna, Austria; 5 Institute of Experimental and Clinical Pharmacology, Medical University of Graz, Graz, Austria; 6 Division of Pulmonology, Department of Internal Medicine, Medical University of Graz, Graz, Austria; 7 Ludwig Boltzmann Institute for Lung Vascular Research, Graz, Austria; University of Giessen Lung Center, Germany

## Abstract

Chronic thromboembolic pulmonary hypertension (CTEPH) is a rare disease with persistent thrombotic occlusion or stenosis of the large pulmonary arteries resulting in pulmonary hypertension. Surgical removal of the neointimal layer of these vessels together with the non-resolved thrombus consisting of organized collagen-rich fibrotic areas with partly recanalized regions is the treatment of choice (pulmonary endarterectomy, PEA). The present study investigates endothelial cells isolated from such material as well as factors present in the surgical PEA material, which may contribute to impairment of recanalization and thrombus non-resolution. We observed muscularized vessels and non-muscularized vessels in the PEA material. The isolated endothelial cells from the PEA material showed significantly different calcium homeostasis as compared to pulmonary artery endothelial cells (hPAECs) from normal controls. In the supernatant (ELISA) as well as on the tissue level (histochemical staining) of the PEA material, platelet factor 4 (PF4), collagen type I and interferon-gamma-inducible 10 kD protein (IP-10) were detected. CXCR3, the receptor for PF4 and IP-10, was particularly elevated in the distal parts of the PEA material as compared to human control lung (RT-PCR). PF4, collagen type I and IP-10 caused significant changes in calcium homeostasis and affected the cell proliferation, migration and vessel formation in hPAECs. The presence of angiostatic factors like PF4, collagen type I and IP-10, as recovered from the surgical PEA material from CTEPH patients, may lead to changes in calcium homeostasis and endothelial dysfunction.

## Introduction

Chronic thromboembolic pulmonary hypertension (CTEPH) is a rare and late complication of venous thromboembolism [1], [2] leading to occluded pulmonary arteries and vascular remodelling [3]. The diagnosis is typically made in advanced stages of the disease when pulmonary vascular resistance is 5–10-fold elevated. Depending on the localization and extent of proximal thrombotic material, a pulmonary endarterectomy (PEA) may be necessary [4]. Between 1 and 5% of patients who survived symptomatic acute pulmonary thromboembolism develop CTEPH [5]. It has been suggested that the reason for the development of the persistent occlusion of the pulmonary artery is a misguided thrombus resolution triggered by infection [6], inflammation [7], autoimmunity, malignancy [8] and/or endothelial dysfunction due to high presence of phospholipid antibodies and lupus anticoagulants [9], [10] rather than prothrombotic factors.

The reason for the incomplete resolution of the clot is currently unknown, but an increased resistance to endogenous thrombolysis due to a polymorphism affecting the fibrinogen α-α chain crosslinkage could be one explanation [11], [12]. Another hypothesis suggests that the differentiation of adventitial fibroblasts or mesenchymal progenitor cells present in the neointima of the occluded vessels of CTEPH patients might be triggered by factors present in the microenvironment of the clot [13]. The myofibroblast- and progenitor cell-rich microenvironment in the pulmonary endarterectomy (PEA) tissue is thought to extensively contribute to the vascular lesion/clot [13], [14]. It is well known that factors from the microenvironment, for example thrombin, potently affect endothelial cells (EC) leading to mobilization of Ca^2+^, rearrangements of the cytoskeleton and endothelial dysfunction [15], [16]. Sakao et al. suggested that the microenvironment created by the unresolved clot in CTEPH patients leads to dysfunctional ECs contributing to the progression of CTEPH [17]. Collagen-secreting cells were detected in PEA material participating in formation of this microenvironment [18].

In CTEPH, partial recanalization of the pulmonary arteries occurs and endothelialized blood vessels may be found in the distal part inside the clot [18], [19]. In vascular systems that are able to form collaterals, the formation of new vessels is regulated by a local balance of pro- and anti-angiogenic factors [20]. Under certain conditions such as tumor formation or wound healing, the positive regulators of angiogenesis predominate. Endothelial cells proliferate, migrate and form a vessel, which is finally stabilized by pericytes and smooth muscle cells [21]. However, angiogenesis in the pulmonary arteries depends on vasa vasorum stemming from the systemic bronchial arteries. After pulmonary arterial occlusion, these vessels spread into the pulmonary arteries and pre-existing collaterals are opened, preventing pulmonary infarction in most of the cases [22]. In CTEPH patients, the number of pulmonary adventitial vasa vasorum increases and the core of the nonresolving clots is recanalized by neovascular endothelialized structures originating from the vasa vasorum [22], [23]. If angiostatic factors (e.g. angiostatin, endostatin, thrombospondin, CXC chemokines lacking ELR motif [20]) outweigh the angiogenic molecules such as VEGF, FGF, angiopoietins, or integrins, angiogenesis may not occur [21], [24]. Numerous soluble growth factors and inhibitors, cytokines and proteases as well as extracellular matrix proteins and adhesion molecules tightly control this multi-step process [21].

**Figure 1:**
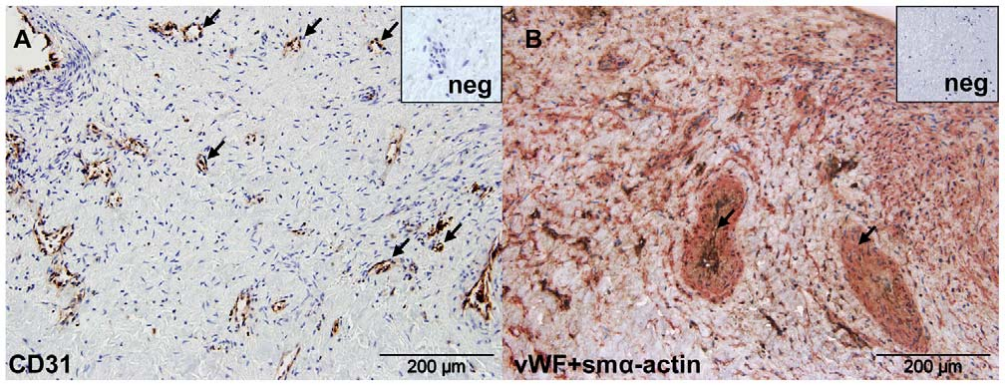
Surgical PEA material contains vessels. (A) CD31 positive endothelial cells (brown) in distal PEA material. (B) Double staining for von Willebrand factor (brown) and smooth muscle α-actin (red) shows muscularized vessels (black arrows). The nuclei are counterstained with Haemalaun (blue). Negative controls are shown in upper right insets.

**Table 1:** Patient characteristics (mPAP – mean pulmonary arterial pressure, PVR – pulmonary vascular resistance, CO – cardiac output, CI – cardiac index).

	CTEPH (n = 38)
**Age (years)**	55 (21−78)
**Gender (female, %)**	40
**mPAP preoperative (mmHg)**	53±13.6
**PVR preoperative (dyn·sec·cm^−5^)**	923±396
**CO preoperative (L·min^−1^)**	4.4±0.83
**Cl preoperative (L·min^−1^·m^−2^)**	2.2±0.3

The role of angiostatin, endostatin and thrombospondin in endothelial cells have already been extensively studied [25]–[27]. Therefore, in the present study investigated the effects of angiostatic factors (collagen, platelet factor 4 (PF4 or CXCL4) and interferon-gamma-inducible 10 kD protein (IP-10 or CXCL10) on the function of endothelial cells isolated from the surgical PEA material. We found high levels of the angiostatic factors in the material and show that these factors cause endothelial dysfunction in control human pulmonary artery endothelial cells.

**Figure 2:**
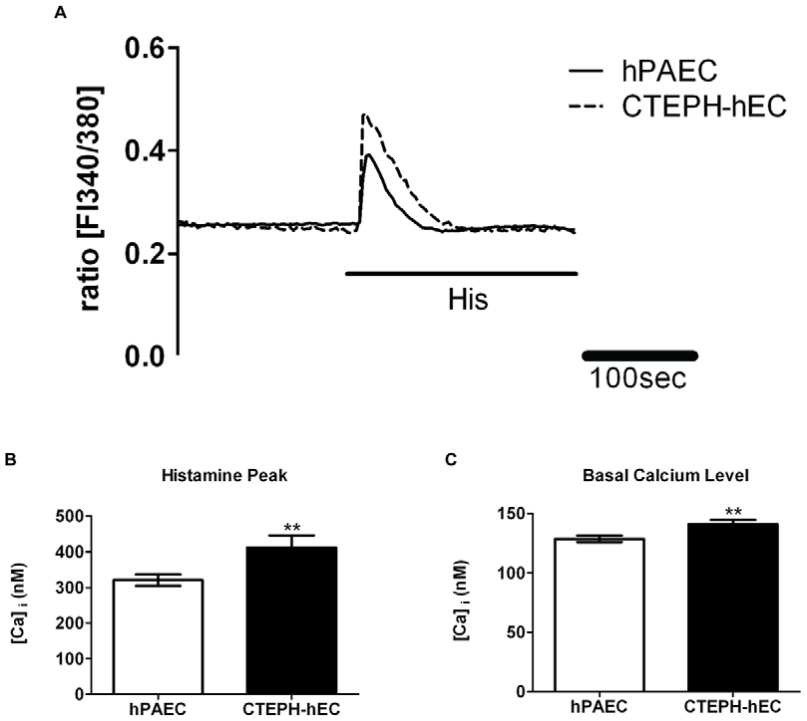
Enhanced calcium handling of CTEPH-hECs compared to hPAECs. (A) Representative graph of the histamine-induced increase in the intracellular calcium concentration. (B) Quantitative data of the histamine-induced peak and (C) basal calcium level in CTEPH-hECs and hPAECs (number of analysed cells n = 132–518). (** p<0.01, *** p<0.001 compared to control hPAECs).

**Figure 3:**
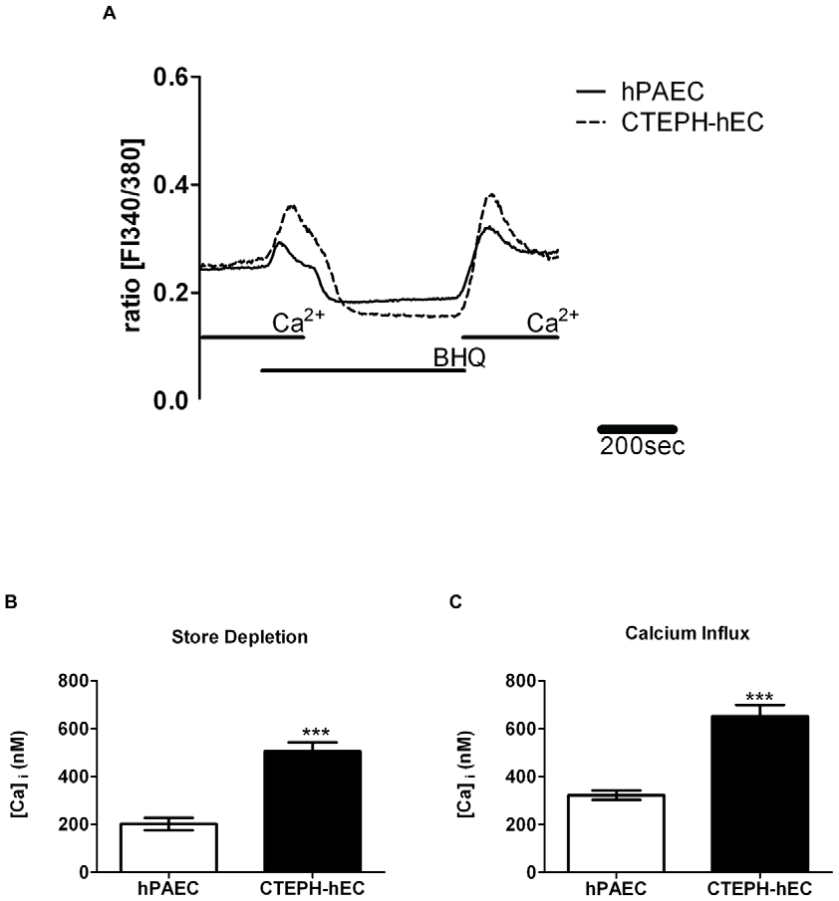
Enhanced store depletion and calcium influx of CTEPH-hECs compared to hPAECs. (A) Representative recording of the calcium store depletion (BHQ = 1,4-dihydroxy-2,5-di-tert-butylbenzene). (B) Quantitative data of the store depletion (measured as the peak of the calcium release) and (C) calcium influx after readmission of calcium in the medium in CTEPH-hECs in comparison with hPAECs (number of analysed cells n = 283–293; *** p<0.001 compared to control hPAECs).

**Table 2:** Calcium parameters of hPAECs vs CTEPH-hECs.

	hPAECs	CTEPH-hECs
**Basal Calcium Level (nM)**	129±3 (n = 518)	141±4 ** (n = 433)
**Histamine Peak (nM)**	376±22 (n = 175)	645±44 *** (n = 132)
**Store Depletion (nM)**	266±21 (n = 283)	375±18 *** (n = 293)
**Calcium Influx (nM)**	408±20 (n = 283)	505±20 *** (n = 293)

## Results

### Vessels are present in surgical PEA material of chronic thromboembolic pulmonary hypertension (CTEPH) patients

The surgical material obtained from the CTEPH patients underwent PEA (Table 1) had an organized, but heterogeneous structure. Distal areas of the material were highly organized, whereas the proximal areas, where sometimes fresh thrombotic material accumulated, were predominantly fibrotic and the cell density was low. In this heterogenous material, the formation of vessel-like structures was observed in the distal areas (Fig. 1 black arrows). These structures included both von Willebrand factor (vWF)^+^ endothelial cells surrounded by smooth muscle α-actin (smα-actin)^+^ cells forming muscularized vessels (Fig. 1B), as well as non-muscularized vessels (in Fig. 1A shown as CD31^+^ cells).

**Figure 4:**
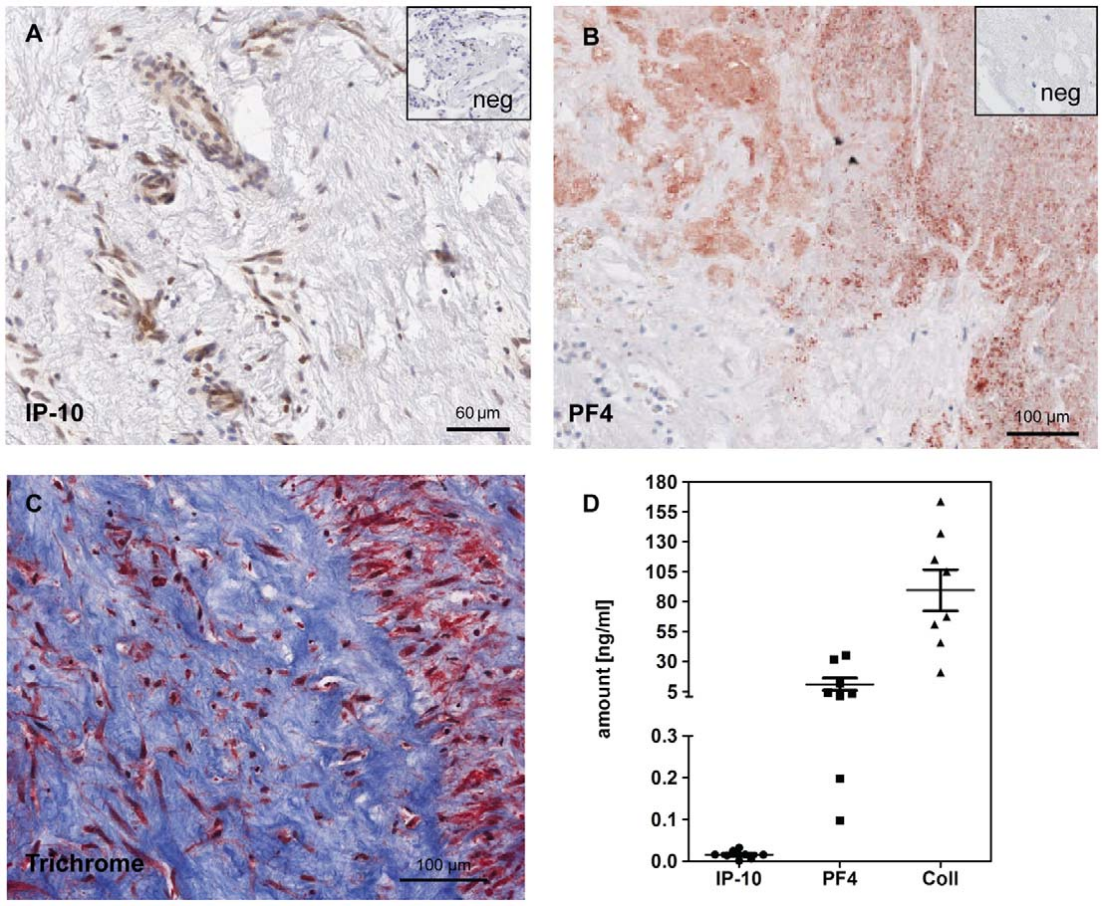
PF4, collagen type I and IP-10 present in PEA material. Immunohistochemical stainings for PF4 (A), trichrome staining (B) and IP-10 (C). Negative controls are shown in the upper right insets. (D) Quantitative data of the amounts of IP-10, PF4 and collagen type I in the supernatant of PEA tissue obtained from 8 different patients.

### Increased intracellular calcium (Ca^2+^) response in endothelial cells from the surgical PEA material (CTEPH-hECs)

A mixed culture of cells was further purified by means of endothelial-specific CD31 magnetic beads. The purified CTEPH-hECs were positively stained for vWF and vascular endothelial (VE) cadherin, but were negative for smα-actin, showing the purity of the culture (Figure S1).

**Figure 5:**
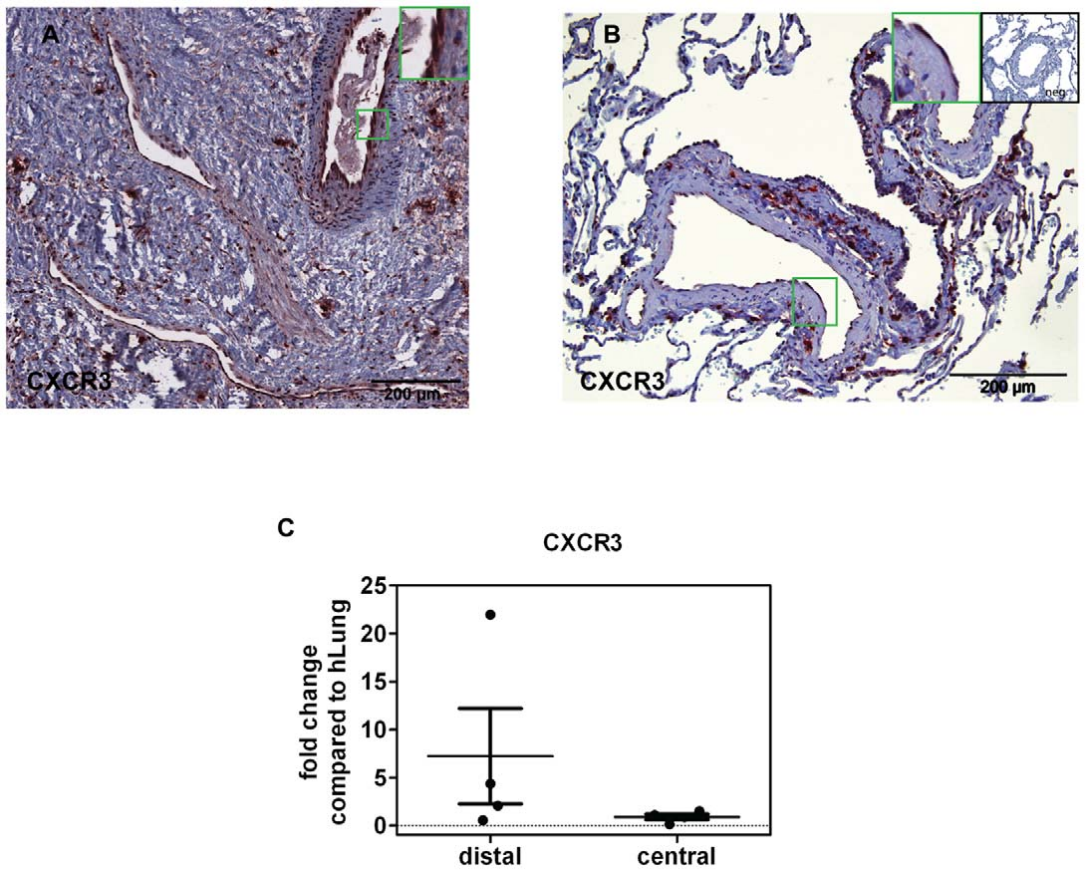
CXCR3 (receptor for IP-10 and PF4) present in PEA material and human lung. (A) Immunohistochemical staining of CXCR3 on paraffin-embedded PEA material. (B) Human lung tissue from a healthy control stained for CXCR3. Green frames show zoom-in regions, negative control is shown in upper right inset. (C) Expression of CXCR3 in distal and proximal parts of PEA material as compared to control human lung shown by RT-PCR.

**Figure 6:**
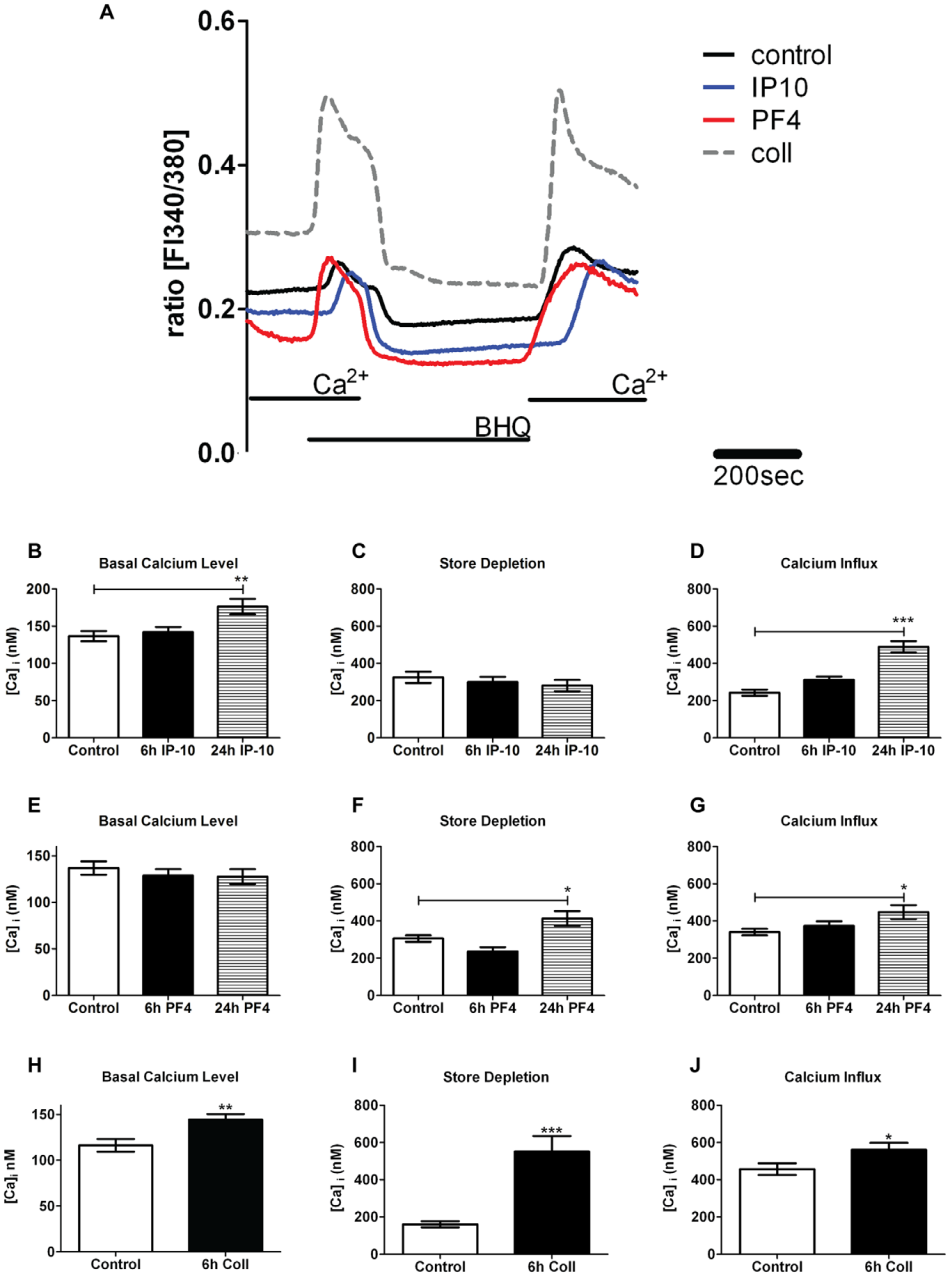
Effect of PF4, collagen type I and IP-10 on calcium (Ca^2+^) handling of hPAECs. (A) Representative Ca^2+^ recording in hPAECs. Bar graphs show the basal Ca^2+^ levels, store depletion (measured as peak of the Ca^2+^ release) and Ca^2+^influx (measured as mean amplitude of the Ca^2+^ signal when external Ca^2+^ was readmitted) of untreated control cells, and cells treated for 6 h and/or 24 h with, (B) 400 ng/ml of PF4, (C) 100 µg/ml of collagen type I and (D) 600 ng/ml of IP-10 (Number of analyzed cells n = 35–109; * p<0.05, ** p<0.01, *** p<0.001 compared to control untreated cells).

**Table 3:** Calcium parameters of hPAECs upon PF4 stimuli.

	control	6h PF4	24h PF4
**Basal Calcium Level (nM)**	137±7 (n = 63)	129±7 (n = 52)	128±8 (n = 52)
**Store Depletion (nM)**	305±18 (n = 105)	236±23 (n = 108)	413±40 * (n = 81)
**Calcium Influx (nM)**	341±17 (n = 106)	373±25 (n = 109)	448±37 * (n = 79)

**Table 4:** Calcium parameters of hPAECs upon collagen type I stimuli.

	control	6h Coll
**Basal Calcium Level (nM)**	116±7 (n = 40)	145±6 ** (n = 35)
**Store Depletion (nM)**	161±16 (n = 58)	551±83 *** (n = 68)
**Calcium Influx (nM)**	457±31 (n = 47)	561±37 * (n = 65)

**Table 5:** Calcium parameters of hPAECs upon IP-10 stimuli.

	control	6h IP-10	24h IP-10
**Basal Calcium Level (nM)**	137±7 (n = 67)	142±7 (n = 47)	176±10 ** (n = 43)
**Store Depletion (nM)**	325±30 (n = 46)	300±28 (n = 62)	280±31 (n = 75)
**Calcium Influx (nM)**	242±17 (n = 49)	312±18 (n = 63)	489±31 *** (n = 76)

Since Ca^2+^ homeostasis affects endothelial function, the basal intracellular Ca^2+^ concentration of CTEPH-hECs was investigated next (Fig. 2A, 3A). The CTEPH-hECs showed a significant increase in the basal intracellular calcium level compared to control human pulmonary artery endothelial cells (hPAECs) (p<0.01, Table 2; Fig. 2C). Histamine challenge resulted in an increased intracellular Ca^2+^ response in CTEPH-hEC as compared to hPAECs (p<0.001; Table 2; Fig. 2B). When the calcium stores of the cells were emptied, in the CTEPH-hECs a significantly increased amount of released calcium could be detected in comparison to hPAECs (p<0.001; Table 2; Fig. 3B). The increase upon extracellular calcium readmission showed a higher calcium influx in CTEPH-hECs as compared to hPAECs (p<0.0001; Table 2; Fig. 3C) suggesting changes in the store-operated calcium entry. In summary, CTEPH-hECs were similar to hPAECs with respect to surface markers (vWF; VE-cadherin), but their calcium homeostasis was markedly different.

**Figure 7:**
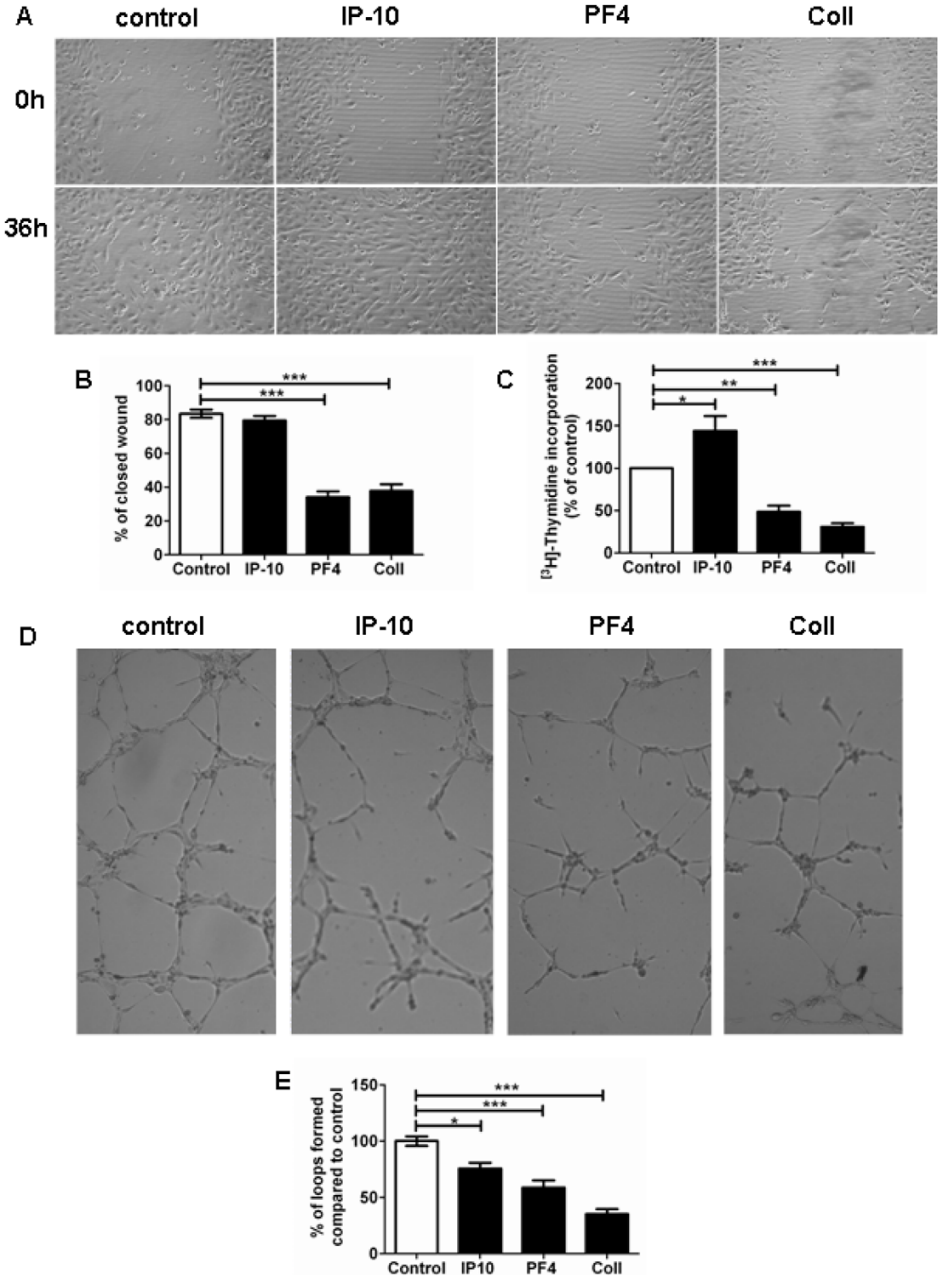
Effect of IP-10, PF4 and collagen type I on hPAEC function. (A) Representative images of migration assay showing the effect of IP-10, PF4 and collagen type I on hPAECs. (B) Quantitative data of wound healing after 36 h (number of experiments n = 3). (C) Bar graph summarizing the effect of these factors on hPAEC proliferation compared to untreated hPAECs (n = 3). (D) Representative image (4X) of vessels formed after 6 h in Matrigel^®^ under different conditions. (E) Bar graph representing the percentage of formed loops compared to control (n = 3) (* p<0.05, ** p<0.01, *** p<0.001 compared to untreated cells).

### Angiostatic factors and their receptors are present in surgical PEA material

The angiostatic factors were determined at the protein level in the supernatant of the PEA tissue. Platelet factor 4 (PF4 or CXCL4) (11±5 ng/ml), collagen type I, (90±17 ng/ml) and interferon-gamma-inducible 10 kD protein (IP-10; 16.1±3.5 pg/ml) were present as detected by ELISA (Fig. 4D). All three factors were also detected at the tissue level by immunohistochemistry (Fig. 4A, B, C). In addition, the receptor for PF4 and IP-10, CXCR3, was found in the PEA surgical material with high expression levels, particularly in the distal areas (Fig. 5A), as compared with healthy lung tissue (Fig. 5B). CXCR3 expression at the mRNA level was likewise higher in CTEPH tissue from the distal parts of PEA as compared with healthy lungs (Fig. 5C). CXCR3 was also present in isolated CTEPH-hECs and hPAECs (Figure S2). CXCR2 mRNA served as an internal control showing no differences in gene expression compared to control lung (data not shown).

### Angiostatic factors and their receptors affect calcium homeostasis

Due to the strong presence of PF4 and collagen type I in the PEA tissue, we investigated their effects on the basal calcium level, store depletion and calcium influx in hPAECs (Table 3 and 4, Fig. 6A). In PF4-stimulated cells (400 ng/ml) neither 6 h nor 24 h of treatment led to a significant change in basal calcium compared to untreated cells (Table 3, Fig. 6B). However, after 24 h, PF4 treatment resulted in a significant increase in calcium store depletion when compared to control cells (p<0.05; Table 3; Fig. 6B). Similarly, PF4 resulted in an increased store-operated calcium entry compared to control after 24 h (p<0.05; Table 3; Fig. 6B). Collagen type I treatment (100 µg/ml) resulted in a significant elevation in basal calcium compared to untreated cells (p<0.01; Table 4; Fig. 6C). Collagen type I also resulted in a significant increase in store depletion (p<0.0001; Table 4; Fig. 6C) and a significantly elevated calcium influx (p<0.05; Table 4; Fig. 6C). This effect was similar to that of PF4. IP-10 treatment (600 ng/ml) changed the basal calcium level significantly compared to control cells only after 24 h (p<0.01; Table 5; Fig. 6D), whereas store depletion showed no difference (Table 5; Fig. 6D). IP-10 treatment increased calcium influx after 24 h significantly compared to control (p<0.001; Table 5; Fig. 6D).

### Angiostatic factors lead to endothelial dysfunction

The effects of IP-10 (600 ng/ml), PF4 (400 ng/ml) and collagen type I (100 µg/ml) on hPAECs were further investigated by means of migration assay (Fig. 7A, B). PF4 treatment resulted in a significantly reduced migration (34±3%), similar to collagen type I treatment (38±4%). Interestingly, treatment with IP-10 did not induce alteration in wound closure compared to untreated cells (80±3% vs. 84±2%; Fig. 7B).

Similarly, PF4 (1000 ng/ml) and collagen type I (100 µg/ml) treatment induced a significant decrease in proliferation compared to untreated cells (29±7% and 31±5%; Fig. 7C). In contrast, IP-10 (600 ng/ml) increased the hPAEC proliferation. Loop formation, tested with an *in vitro* angiogenesis assay, was significantly decreased by all treatments (IP-10: 76±5%; PF4: 59±6%; collagen type I: 35±4%) as compared to control. The strongest effect was observed in the collagen type I-treated hPAECs (Fig. 7D, E).

## Discussion

Chronic thromboembolic pulmonary hypertension (CTEPH) is a rare and late complication of venous thromboembolism. A fresh pulmonary embolus normally gets dissolved by fibrinolysis [28], macrophages [29]–[31] and by recanalization [32], [33]. In CTEPH patients, the recanalization does not occur or is incomplete [17], [18], [34] resulting in elevation of pulmonary vascular resistance and secondary remodeling of non-occluded vessels.

In our study, we investigated endothelial cells from surgical PEA material (CTEPH-hECs), compared them with control hPAECs and tested the effect of mediators secreted by the PEA material on hPAECs. Our readouts included calcium homeostasis, proliferation, migration and vessel formation. Similar to previous studies [18], [19], [34], [35], we observed newly formed vessels in the surgical PEA material. This neovascularization/ recanalization has been suggested to be due to endothelial progenitor cells and/or hPAECs and pulmonary artery smooth muscle cells (PASMCs) migrating from the pulmonary artery or from the systemic circulation and being trapped in the thrombotic clot [3], [18], [36].

Calcium is a key regulatory molecule for endothelial function [37]. We observed a significant rise in the basal calcium level of the CTEPH-hECs as compared to the hPAECs. Furthermore, in our study histamine challenge led to a stronger response in the CTEPH-hECs as compared to hPAECs. We applied a calcium ATPase inhibitor to induce depletion of the calcium stores and to identify the reason for this calcium rise. By measuring the readmission of the external calcium, the store-operated calcium influx was quantified and compared to healthy control cells. With this tool, possible dysfunctions either in calcium channels of the plasma membrane or in the calcium stores can be detected. The increased calcium depletion of the CTEPH-hECs compared to hPAECs showed that these cells accumulate more calcium in their stores, which is a sign of altered calcium homeostasis. The readmission of external calcium was greater in the CTEPH-hECs compared to control cells. One possible reason for this increase could be the higher expression or sensitivity of transient receptor potential canonical (TRPC) channels in the plasma membrane. Increased TRPC expression has been previously described in PASMCs isolated from patients with idiopathic pulmonary arterial hypertension (IPAH) [38].

Beside the altered calcium homeostasis, CTEPH-hECs showed a typical endothelial phenotype: cobblestone monolayer formation, expression of vWF and continuous VE-cadherin membrane staining. No significant differences in vessel formation and wound closure properties were observed in CTEPH-hECs compared to the hPAECs (Figure S3). A recent study reported that isolated EC-like cells from surgical PEA material exhibit a defective mitochondrial structure, inability to form autophagosomes and morphologic changes after some passages [17]. The microenvironment present in the PEA material may cause endothelial dysfunction as observed by Sakao et al. [17]. Another study suggested that this microenvironment caused hyperproliferation, invasiveness, and anchorage-independent growth of myofibroblasts [39]. In our study, we identified three factors which may cause endothelial dysfunction. The expression of PF4, collagen type I and IP-10 were visualized in the surgical PEA material. All these proteins were quantified in the supernatant of the tissue. PF4 is exclusively expressed in developing megakaryocytes and stored in α-granules of platelets. Activated platelets release PF4 at sites of injury [40], [41]. IP-10 is reported to activate and recruit effector T cells and other leukocytes [42] and is strongly up-regulated in many inflammatory diseases [43]. Finally, collagen type I is important for wound healing and formation of extracellular matrix, which may promote proliferation of smα-actin^+^ cells and differentiation of injured ECs into smα-actin^+^ cells [18]. Taken together, these factors may harbor angiostatic features in the PEA material potentially contributing to the development of CTEPH.

In our study, human pulmonary artery endothelial cells (hPAECs) were challenged with PF4, collagen type I and IP-10 to characterize their effects on calcium homeostasis in order to mimic the microenvironment in the PEA material. Only collagen increased the basal calcium level, while store depletion and calcium influx were increased by collagen type I, as well as by IP-10 and PF4. This suggests that the microenvironment of the surgical PEA material significantly affects the calcium homeostasis of endothelial cells. Fibrin and thrombin have been previously shown to increase basal calcium levels in EC from PEA material [44]. The question, how PF4 and IP-10 may evoke angiostatic effects in hPAECs is still unsolved. PF4 and IP-10 are known to be ligands for CXCR3 [45], [46]. Romagnani et al. showed that human microvascular endothelial cells express CXCR3 which mediates angiostatic effects [46]. In their study, the percentage of CXCR3 positive vessels in diseased tissue was significantly higher compared to healthy tissue. We detected CXCR3 expression in hPAECs as well as in CTEPH-hECs, indicating that its ligands, IP-10 and PF4, might evoke an angiostatic effect by activating CXCR3. PEA material, compared to human lung, showed abundant CXCR3, visualised by immunohistochemistry particularly in the vessels. On the mRNA level, CXCR3 was up-regulated in the distal PEA areas as compared to control lung. In addition, a hallmark for chemokine activation is the ability to initiate calcium signalling [47], mainly via G-protein coupled receptors. It has been reported previously that stimulation of CXCR3 leads to mobilization of intracellular calcium [47 50]. The question, whether IP-10, PF4 and collagen type I act on calcium homeostasis via CXCR3 in CTEPH-hECs needs further investigation.

An important feature of ECs is vessel formation and migration [24]. We observed that PF4 and collagen type I significantly decreased the migration and vessel formation of hPAECs. Furthermore, proliferation was inhibited by both PF4 and collagen treatment, pointing to angiostatic effects of these factors. PF4 is thought to act as an angiostatic factor by interacting with pro-angiogenic molecules such as VEGF or FGF, cell adhesion molecules and integrins [51]–[53]. For example it was shown that PF4 inhibits FGF2 dimerization by complex formation and therefore reduces the binding to and internalization of FGF receptor [54]. In contrast to PF4, in our investigation IP-10 did not decrease the proliferation of hPAECs. This is in line with observations of Angiolillo et al. who showed that IP-10 did not inhibit HUVEC proliferation, but suppressed HUVEC differentiation into capillary structures [55], [56]. However, in HUVECs, HMVECs and cardiac ECs, IP-10 had anti-proliferative effects [43]. PF4, collagen type I and IP-10 significantly decreased the degree of vessel formation in hPAECs in our investigations, pointing to an angiostatic effect on hPAECs. Similarly, PF4 has been shown to inhibit the migration of human endothelial progenitor cells, as well as to suppress microvessel formation in myeloma xenografts [57]. Futhermore, IP-10 has been reported to act as an angiostatic factor *in vivo* [58] and *in vitro* [59]. Our data indicate that all the above factors could interfere with endothelial vessel-forming ability *in vivo*.

In conclusion, we observed altered calcium homeostasis in endothelial cells from surgical PEA material and the presence of angiostatic factors as well as their receptor. Furthermore, our investigations show that angiostatic factors alter the calcium handling in normal hPAECs, leading to the conclusion that the microenvironment in PEA material might contribute to the altered calcium homeostasis in CTEPH-hECs. These observations suggest that IP-10, collagen and PF4 lead to angiostatic effects, shown by decreased angiogenesis and/or proliferation and migration. As a consequence there might be inadequate recanalization of the thromboembolic material in CTEPH.

## Materials and Methods

### Ethics Statement

The study protocol for tissue donation was approved by the Institutional Review Board of the Medical University of Vienna (Ek-Nr: 903/2009) in accordance with national law, and with guidelines on Good Clinical Practice/International Conference on Harmonization. Written informed consent was obtained from each individual patient.

### Surgical pulmonary endarterectomy (PEA) material

The surgical material was obtained from chronic thromboembolic pulmonary hypertension (CTEPH) patients (n = 38) undergoing PEA. The patient characteristics are presented in Table 1. Values are representing mean values ± standard deviation. The surgical material was transported directly from the operating room to the cell laboratory in endothelial culture media (VascuLife® VEGF Cell Culture Medium; LifeLine Technology, Walkersville) and was processed immediately thereafter.

### Isolation of primary human endothelial cells from PEA material

The PEA material was carefully cut into small pieces under microscopic guidance and placed into 75 cm^3^ culture flasks with a drop of endothelial cell (EC) culture media (VascuLife® VEGF Cell Culture Medium; LifeLine Technology, Walkersville). The tissue with the outgrowing cells was maintained at 37°C and 5% CO_2_. After 24 h the media was filled up to 15 ml and then changed every 48 h. For purifying the EC culture, magnetic bead sorting with CD31 antibodies (91935, Milteny) on MACS® was performed using the POSSEL program. The identity of purified CTEPH-hECs was verified by their characteristic appearance in phase-contrast microscopy, followed by immunocytochemical stainings for von Willebrand factor (vWF) (dilution 1∶100; Dako) and vascular–endothelial cadherin (VE-cadherin) (1∶200, Santa Cruz Biotechnologies).

### Immunohistochemistry

Fresh surgical material from pulmonary endarterectomy was fixed in 4% formaldehyde for 24 h and embedded in paraffin blocks. The sliced paraffin tissue (2 µm thick) was placed on Capillary Gap Microscope Slides (Dako REAL^TM^) and kept for further use at room temperature. Single or double staining was carried out with primary antibodies against vWF (dilution 1∶100; Dako) and/or smα-actin (dilution 1∶100; Sigma) and goat-anti-rabbit-HRP (dilution 1∶100; Santa Cruz Biotechnologies) or goat-anti-mouse-HRP (dilution 1∶100; Santa Cruz Biotechologies) secondary antibodies. For detection DAB (Dako) was used. For CD31 staining (dilution 1∶100; Abcam) the DAB detection kit from R&D Systems was applied. The immunhistochemical stainings for CXCR3 (1∶250, ab64714), IP-10 (1∶250; ab9807) or PF4 (1∶250; ab49735) were performed with the same kit. For the collagen fibre staining the Massońs Trichrome staining protocol was used, according to the manufacturer's instructions.

### Live cell calcium (Ca^2+^) imaging

CTEPH-hECs from six patients and human pulmonary artery endothelial cells (hPAECs; CC-2530) from three donors were cultured on 25 mm diameter, gelatin-coated glass cover slides until confluence. After incubation the cells were loaded for 45 minutes with 2 μM fura-2/AM and washed with Ringer solution (5,8 mM KCl, 141 mM NaCl, 0,5 mM KH_2_PO_4_, 0,4 mM NaH_2_PO_4_, 11,1 mM glucose, 10 mM Hepes, 1,8 mM CaCl_2_, 1 mM MgCl_2_, pH 7,4). After 25 minutes the single glass cover slide was mounted on the stage of a Zeiss 200 M inverted epifluorescence microscope coupled to a PolyChrome V monochromator (Till Photonics) light source in a sealed, temperature-controlled RC-21B chamber (Warner Instruments). Fluorescence images were obtained with alternate excitation at 340 and 380nm. Emitted light was collected at 510 nm by an Andor Ixon camera. The acquired images were stored and subsequently processed offline with TillVision software (Till Photonics). During the measurement the cells were perfused with 10 μM histamine or 15 μM 1,4-dihydroxy-2,5-di-tert-butylbenzene (BHQ: selective SERCA blocker) in the presence or absence of extracellular calcium.

Measurements were made every 3s. Background fluorescence was recorded from each cover slip and subtracted before calculation. Maximal and minimal ratio values of the [Ca^2+^]_i_ were determined at the end of each experiment by first treating the cells with 1 µmol/L ionomycin (maximal ratio) and then chelating all free Ca^2+^ with 10mmol/L EGTA (minimal ratio). Cells that did not respond to ionomycin were discarded. [Ca^2+^]_i_ was calculated as described earlier [60], [61].

For measuring the effect of the microenvironment, the hPAECs were pre-treated for 6 h and 24 h with 400 ng/ml PF4 (Peprotech), 600 ng/ml IP-10 (Peprotech), or 100 µg/ml collagen type I (Sigma) or left untreated (control).

For data analysis, the basal level of Ca^2+^ was determined as an average value of the first 50 seconds of the curve. The agonist-induced Ca^2+^ response was calculated as the peak height subtracting the baseline. The BHQ-induced Ca^2+^ peak height after subtracting the baseline as well as the Ca^2+^ response upon external Ca^2+^ readmission were quantified.

### Proliferation

To investigate the effect of different stimuli on hPAECs the following protocol was applied: 5.000 hPAECs (from three different donors) were seeded in 96 well plates, the following day the cells were treated for24 h with 600 ng/ml IP-10 (Peprotech), 1000 ng/ml PF4 (Peprotech) 100 µg/ml collagen type I (Sigma) or kept under control conditions (VascuLife® Basal Medium with 2%FCS; LifeLine Technology, Walkersville). The growth and proliferation of hPAECs was determined by [^3^H]-thymidine (BIOTREND Chemikalien GmbH) incorporation as an index of DNA synthesis and measured as radioactivity by a scintillation counter (Wallac 1450 MicroBeta TriLux Liquid Scintillation Counter & Luminometer). Experiments were performed in quintuplicates.

### Determination of protein amount in the supernatant of PEA tissue

After removal, the PEA samples from CTEPH patients (n = 8) were immediately put in endothelial cell basal medium supplemented with 5% FCS and antibiotics (VascuLife® VEGF Cell Culture Medium; LifeLine Technology, Walkersville). The tissue was cut into small pieces and incubated for 18h at 37°C in DMEM-F12 medium (Gibco) with 0.3% FCS, glutamine, penicillin and streptomycin (1% each). The supernatant was collected and stored at −80°C; afterwards the tissue was briefly washed with Hepes buffer and lysed in 150 µl RIPA buffer with PhosphoStop and Protease Inhibitor Complex. IP-10 contents in the supernatant were determined using the IP-10 Flex set and a FACS Canto II flow cytometer (Becton Dickinson, Franklin Lakes, NJ).

For the quantitative measurement of PF4 and collagen type I in the supernatant of PEA tissue we used enzyme-linked immunosorbant assays (ELISA; from RayBio and Cosmo Bio, respectively) according to the manufacturers' instructions.

### RNA-isolation and PCR

Total cellular RNA from hPAECs of three donors was isolated with the RNeasy Mini Kit from Qiagen. For RNA isolation from PEA material and human lungs of four patients each, the Trizol protocol was applied. A Nanodrop 2000c spectrophotometer (PeQlab) was used to quantify the concentration and the purity of the isolated total RNA. The total RNA was converted to cDNA using a RevertAid H Minus First Strand cDNA Synthesis kit (Fermentas). PCR amplifications were performed by a Lightcycler 480 (Roche).

### Migration assay

Human PAECs of three donors were cultured until confluence in culture inserts (Ibidi) for 24-well plates. By removing the inserts a wound of approximately 400 µm±50 µm was made. The cells were than stimulated either with 600 ng/ml IP-10, 400 ng/ml PF4 or 100 µg/ml collagen type I for 36 h or kept in control conditions (EC-culture media) (t_0_). The migrating cells were monitored in a Zeiss Cell Observer (Carl Zeiss inc., Germany) and pictures were taken every 2 h for 72 h with a 10X objective. The results were analyzed via measuring the actual wound size at a given time point (t_36h_) and normalizing it to the 0 h size. Experiments were performed three times in duplicates.

### Vessel formation assay

The In Vitro Angiogenesis Assay Kit (ECM625, Millipore) was used to test the effect of 600 ng/ml IP-10, 400 ng/ml PF4, and 100 µg/ml collagen type I in ECs. Matrigel^®^ was prepared in a 96 well plate according to the manufactureŕs instructions and 16.000 hPAECs of three donors were seeded in each well. After 6 h of stimulation, phase contrast pictures were taken with a light microscope (Zeiss epifluorescent 4X). To compare the vessel formation ability of CTEPH-hECs and hPAECs, 16.000 cells of three donors were seeded on Matrigel^®^. After 21 h, pictures were taken with a light microscope (Zeiss epifluorescent 4X). The formed loops were counted and compared to control cells (cultured only in full media). Experiments were performed three times in triplicates.

### Statistical analysis

Numerical values are given as means ± SEM of n cells or measurements. Intergroup differences were assessed by factorial analysis of variance with post hoc Bonferroni test or Studentś unpaired t-test as appropriate. p-values <0.05 were considered significant and are shown as * in the figures and tables (* p<0.05, ** p<0.01, *** p<0.001).

## Supporting Information

Figure S1 **Morphological characterization of endothelial cells from surgical PEA material.** (A) Mixed culture of cells growing out from the PEA material. Significant proportion of cells show vWF positive signal (brown). (B) Purified, vWF (brown) positive endothelial culture after CD31 magnetic bead sorting. (C) Lack of the staining for smooth muscle α-actin in the CD31-sorted CTEPH-hEC population. (D) CTEPH-hECs form a tight monolayer with intense VE-cadherin (red) staining. Insets show the negative controls. Nuclei are counterstained blue with DAPI.
(TIF)

Figure S2 **Presence of CXCR3 mRNA in tissue and endothelial cells.** Expression of CXCR1 (lane 1), CXCR2 (lane 2), CXCR3 (lane 3), CXCR5 (lane 5) and B2M shown in PEA tissue, human lung (A), CTEPH-hECs and hPAECs (B) as shown by PCR.
(TIF)

Figure S3 **Migration and vessel formation ability of CTEPH-hECs and hPAECs.** (A) Representative image (4X) of vessel formation on Matrigel^®^ after 21h of CTEPH-hECs and hPAECs. (B) Bar graph shows summarized results of vessel formation assay as number of loops formed (number of experiments n = 3). (C) Representative images of migration assay of CTEPH-hECs and hPAECs. (D) Graph shows normalized results of the migration assay as decrease in cell free area after 48 h (n = 3) (* p<0.05, ** p<0.01, *** p<0.001 compared to control untreated cells).
(TIF)
