# Supplementary figures and images for: Angiostatic Factors in the Pulmonary Endarterectomy Material from Chronic Thromboembolic Pulmonary Hypertension Patients Cause Endothelial Dysfunction
Source: PLoS One. 2012 Aug 20;7(8):e43793. doi: 10.1371/journal.pone.0043793 (PMC3423379; doi:10.1371/journal.pone.0043793)

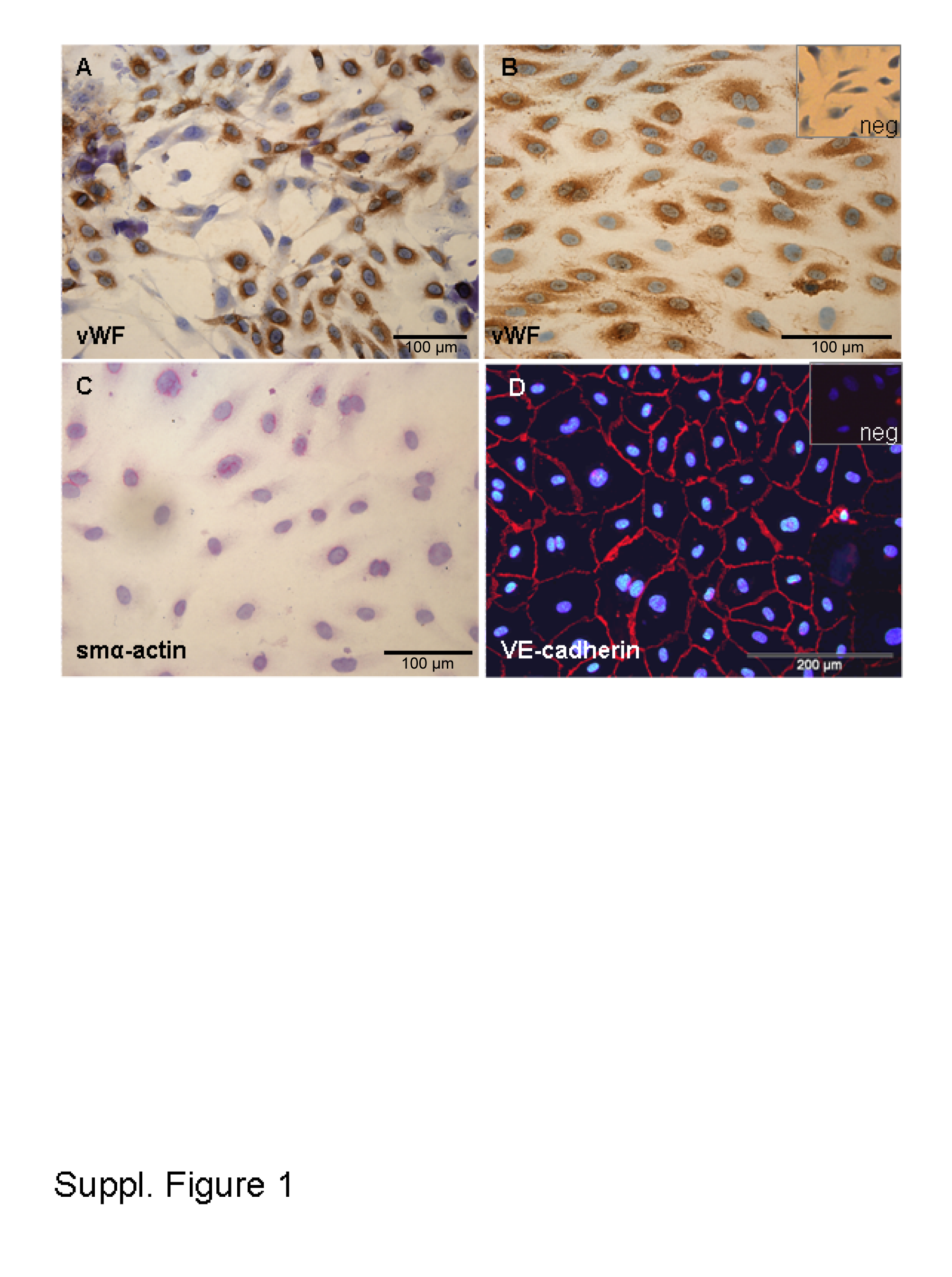

Supplement: Figure S1 — Morphological characterization of endothelial cells from surgical PEA material. (A) Mixed culture of cells growing out from the PEA material. Significant proportion of cells show vWF positive signal (brown). (B) Purified, vWF (brown) positive endothelial culture after CD31 magnetic bead sorting. (C) Lack of the staining for smooth muscle α-actin in the CD31-sorted CTEPH-hEC population. (D) CTEPH-hECs form a tight monolayer with intense VE-cadherin (red) staining. Insets show the negative controls. Nuclei are counterstained blue with DAPI. (TIF) [file pone.0043793.s001.tif]

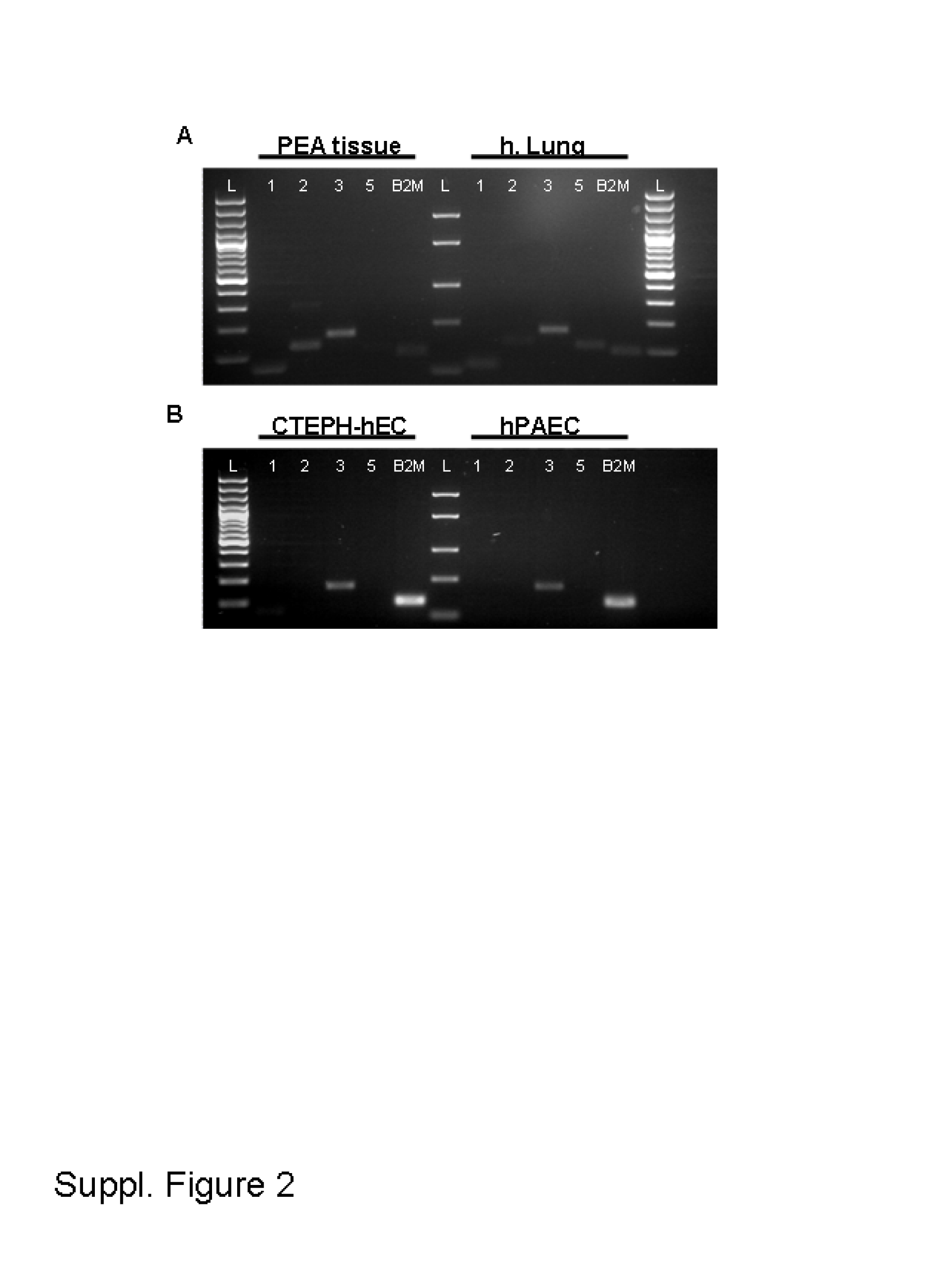

Supplement: Figure S2 — Presence of CXCR3 mRNA in tissue and endothelial cells. Expression of CXCR1 (lane 1), CXCR2 (lane 2), CXCR3 (lane 3), CXCR5 (lane 5) and B2M shown in PEA tissue, human lung (A), CTEPH-hECs and hPAECs (B) as shown by PCR. (TIF) [file pone.0043793.s002.tif]

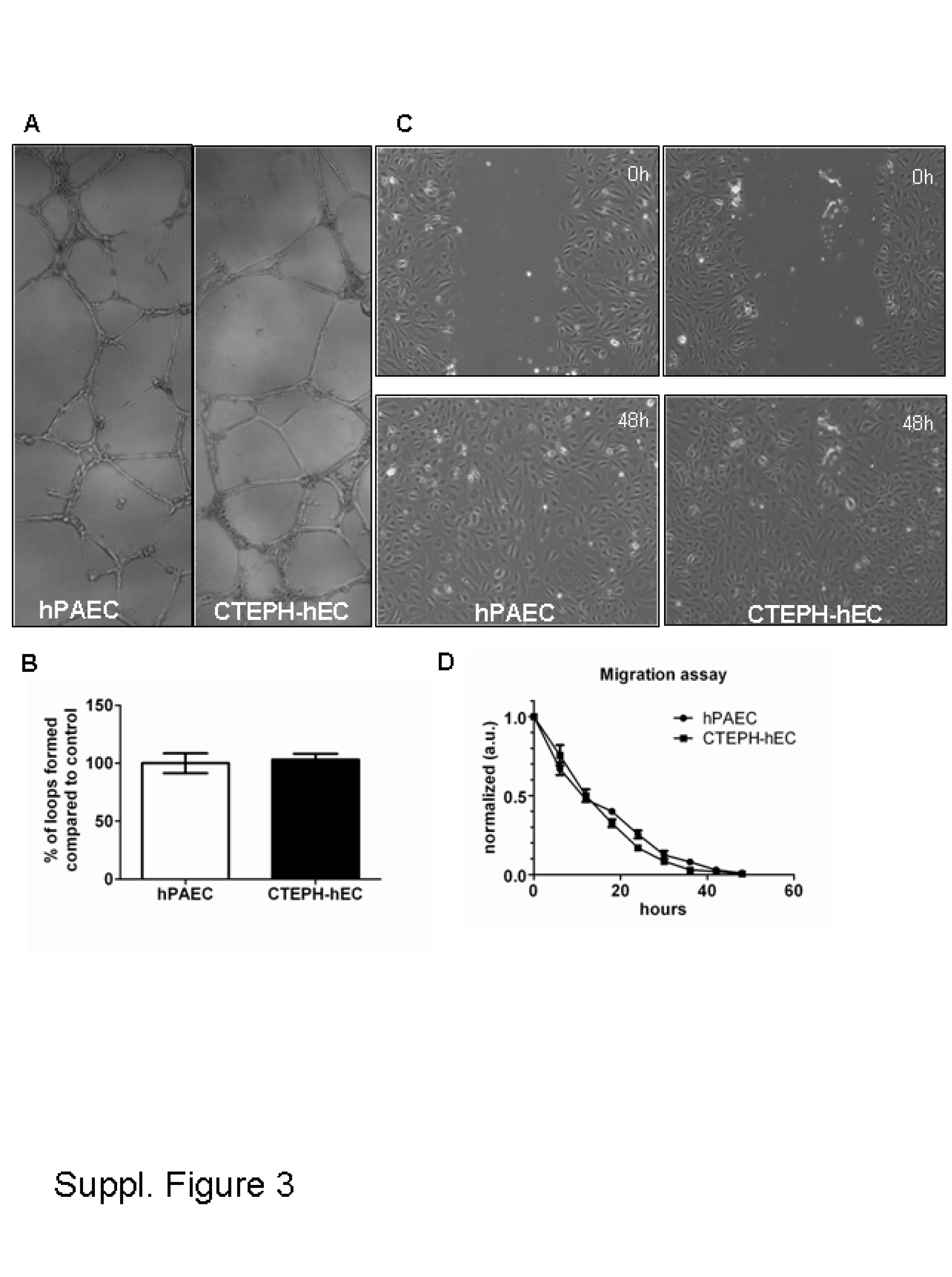

Supplement: Figure S3 — Migration and vessel formation ability of CTEPH-hECs and hPAECs. (A) Representative image (4X) of vessel formation on Matrigel® after 21h of CTEPH-hECs and hPAECs. (B) Bar graph shows summarized results of vessel formation assay as number of loops formed (number of experiments n = 3). (C) Representative images of migration assay of CTEPH-hECs and hPAECs. (D) Graph shows normalized results of the migration assay as decrease in cell free area after 48 h (n = 3) (* p<0.05, ** p<0.01, *** p<0.001 compared to control untreated cells). (TIF) [file pone.0043793.s003.tif]
